# Are U.S. food and beverage companies now advertising healthy products to children on television? An evaluation of improvements in industry self-regulation, 2017–2021

**DOI:** 10.1186/s12966-023-01517-y

**Published:** 2023-10-04

**Authors:** Melissa L. Jensen, Frances Fleming-Milici, Jennifer L. Harris

**Affiliations:** 1 UConn Rudd Center for Food Policy and Health, University of Connecticut, One Constitution Plaza Suite 600, Hartford, CT 06103 USA; 2 School of Nutrition, University of Costa Rica, San José, Costa Rica

**Keywords:** Food marketing, Food industry, Public health, Obesity prevention

## Abstract

**Background:**

Through the Children’s Food and Beverage Advertising Initiative (CFBAI), U.S. food companies pledge to only advertise healthier products in children’s television (TV) programming, but previous research shows that highly advertised products do not qualify as nutritious according to independent nutrition criteria. In 2020, the CFBAI implemented stricter nutrition criteria for products that may be advertised to children, but the potential impact of these changes has not been assessed. This observational study evaluates (1) improvements in energy and individual nutrient composition of products that companies indicated may be advertised to children (i.e., CFBAI-listed products) in 2020 versus 2017, (2) amount of advertising on children’s TV for CFBAI-listed versus other products in 2021, and 3) the nutrition quality of advertised versus non-advertised CFBAI-listed products.

**Methods:**

Data include energy, saturated fat, sodium, and sugar content and overall nutrition quality (Nutrition Profile Index [NPI] scores) of CFBAI-listed products in 2017 (*n* = 308) and 2020 (*n* = 245). Nielsen data provided total ad spending and children’s exposure to ads on children’s TV channels for all foods and beverages in 2021.

**Results:**

From 2017 to 2021, energy, saturated fat and sugar declined for CFBAI-listed products in three of six food categories (yogurt, sweet and salty snacks). Although CFBAI-listed products accounted for 79% of food ads viewed by children on children’s TV channels, just 50% of CFBAI-listed food and 36% of drink brands were advertised on children’s TV. Moreover, advertised products were significantly less nutritious than non-advertised CFBAI-listed products.

**Conclusion:**

Despite revised nutrition standards and improvements in nutrient content of some product categories, participating companies continued to primarily advertise nutritionally poor food and beverages on children’s TV. CFBAI companies have not delivered on their promises to advertise healthier products to children.

## Background

Globally, children are exposed to large amounts of food and beverage marketing that primarily promotes products high in fat, sugars and/or sodium, with little nutritional value [1]. Food and beverage marketing increases children’s preferences and consumption of unhealthy versus healthy products, with negative long-term impact on dietary quality and diet-related diseases [2]. Despite limited evidence of effectiveness, industry voluntary self-regulatory programs remain the most common policy mechanism to address the significant public health concerns raised by food and beverage marketing aimed at children. Moreover, industry self-regulatory programs only cover advertising to children under age 12 to 14 years, whereas the World Health Organization (WHO) calls for governments to protect children below age 18 years from unhealthy food marketing [3].

The U.S. food and beverage industry self-regulatory program, the Children’s Food and Beverage Advertising Initiative (CFBAI), was launched in 2006 with the goal of “shifting the mix” of foods and beverages advertised to children under 12 years to encourage heathier dietary choices. As of 2022, 20 of the largest U.S. food and beverage companies participated in CFBAI and pledged to only advertise products that meet CFBAI nutrition criteria in child-directed media (defined as media with a high proportion of children under 12 years in the audience) or to not advertise any products in child-directed media [4]. Most participating companies also have pledged they will not advertise any products in media primarily directed to children under 6 years. The CFBAI regularly publishes a list of products that meet its nutrition standards and that companies indicate may be advertised in child-directed media (i.e., CFBAI-listed products). The products and brands on these lists change frequently over time, and companies do not advertise all products included on these lists on children’s TV channels [5].

**Fig. 1:**
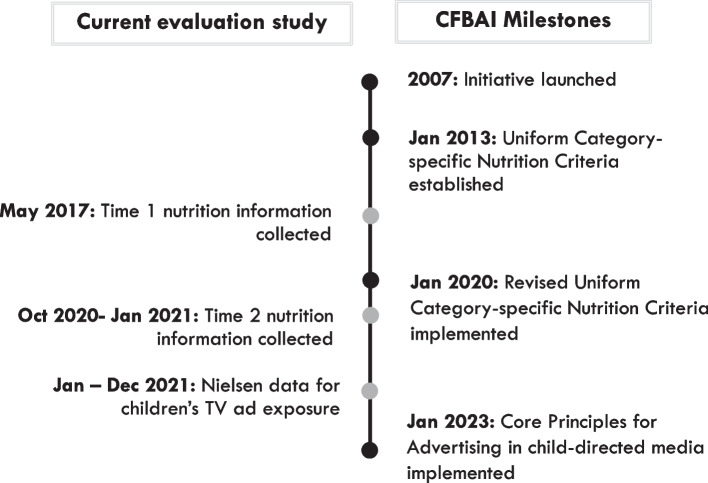
CFBAI program milestones and evaluation timing

In response to critiques by public health experts, the CFBAI has made improvements to the program since its launch (see Fig. 1). For example, effective January 2023, the CFBAI updated its core principles (6^th^ edition) to cover advertising in media primarily directed to children under 13 years but did not change its nutrition criteria [6]. The program has also improved the nutrition criteria it uses to identify “healthier” products that may be advertised in child-directed media. In 2013, the CFBAI introduced category-specific nutrition criteria, which applied uniform criteria for individual food and drink categories across all participating companies. In a study of TV advertising to children (under 12) in 2017 [7], the majority of products advertised on children’s commercial television (TV) channels (e.g., Nickelodeon, Nick Jr., Cartoon Network, Disney XD) by CFBAI-participating companies continued to contain high levels of sugar, fat and/or sodium, including sugary cereals, fruit snacks and other snacks, meal products, and fruit drinks [7, 8]. Moreover, some of the most nutritious products on the CFBAI lists had little or no advertising on children’s TV channels (e.g., yogurts, low-sugar cereals, carrots), whereas the products with the most advertising had some of the lowest overall nutrition scores (e.g., high-sugar cereals, Goldfish crackers). In addition, CFBAI-listed products represented less than one-half of ads viewed by children under age 12 for all CFBAI company brands on all TV programming [7].

The CFBAI revised its nutrition criteria again in 2020 in response to the release of the 2015–2020 Dietary Guidelines for Americans (DGAs) and the new Food and Drug Administration (FDA) nutrition label [9]. These new criteria specify per-serving limits for energy, saturated fat, sodium, and added sugar for individual food and drink categories, and require a specified amount of nutrient components to encourage, including fruit, vegetables, dairy, and/or whole grain, or fortification. A recent study found that overall nutrition quality for CFBAI-listed food products improved for just two individual categories (yogurt and sweet snacks) following introduction of the new criteria (2020 vs. 2017), but not for CFBAI-listed products in total [5]. The proportion of CFBAI-listed beverages with added sugar and/or non-nutritive sweeteners (NNS) also declined, and milk and unsweetened water products were added to the lists in 2020.

However, additional analyses of the individual nutrients of concern for CFBAI-listed products is needed to evaluate CFBAI assertions that its revised nutrient criteria reflect the 2015–2020 DGAs [10]. Moreover, research is needed to understand whether and how changes in CFBAI nutrition criteria have affected the food and beverages that companies actually advertise to children and whether child-directed advertising now promotes healthier options. It is important to understand whether companies have refocused their child-directed advertising to promote categories with healthy nutrient content (e.g., yogurts, unsweetened waters) over their least healthy categories (e.g., sugary cereals, snacks), which in the past have been responsible for the majority of child-directed advertising [7].

The objectives of this study were to (1) examine changes in the energy and individual nutrient content of products that CFBAI companies indicated may be advertised to children (i.e., CFBAI-listed products) following implementation of CFBAI revised nutrition criteria in January 2020; (2) quantify the amount of advertising spending and ads viewed for CFBAI-listed and other products on children’s TV channels (i.e., a primary form of child-directed media) in 2021; and (3) compare the nutrition quality of CFBAI-listed products that were advertised on children’s TV channels (CTV) in 2021 to CFBAI-listed products that were not advertised.

## Methods

This study used a repeated cross-sectional design to assess changes in individual nutrient content, including by product category, of CFBAI-listed products pre- and post-implementation of revised CFBAI nutrition criteria. For these analyses we utilized a database of nutrition data collected in 2017 and 2020 for a previous study [5]. In addition, we obtained Nielsen syndicated market research data, which quantifies advertising spending and the average number of food and beverage advertisements viewed by preschoolers (2–5 y), children (6–11 y), and adults (18–49) on children’s TV channels in 2021, including CFBAI-listed products and other products advertised by CFBAI and non-CFBAI companies. Table 1 lists CFBAI members at the time of the study. Of note, McDonald’s USA and Burger King Corporation were CFBAI members, but the Nielsen analysis did not include any restaurant advertising so we do not report their advertising. Institutional Review Board approval was not required, because the study did not involve human subjects.

**Table 1:** CFBAI participating companies

Companies that committed to advertise only foods that meet CFBAI’s Category-Specific Uniform Nutrition Criteria in child-directed advertising	Companies that committed to not engage in any child-directed advertising
Burger King Corporation	American Licorice Company
Campbell Soup Company	The Coca-Cola Company
Conagra Brands	Ferrero USA
Danone North America	The Hershey Company
General Mills	Keurig Dr Pepper
Kellogg Company	Mars
The Kraft Heinz Company	Mondelez International
McDonald’s USA	Unilever United States
Nestlé USA	
PepsiCo	
Post Foods	

*Source*: *BBB* Children’s Food and Advertising Initiative. CFBAI Product List (August 2021)

### Product sample

The nutrition database included all products on CFBAI Product Lists published in August 2020 (*n* = 245) and January 2017 (*n* = 308). Each product was allocated to one of 19 product categories listed in the CFBAI’s 2020 Revised Nutrition Criteria [11]. The Nielsen data identified all food and beverage brands advertised on children’s TV channels in 2021, including brands from CFBAI participating and non-CFBAI companies.

### Data collection

The database of CFBAI-listed products in 2017 and 2020 included detailed nutrition information obtained from Nutrition Facts labels and ingredient lists on company and/or brand websites, collected May to July 2017 [7] and October 2020 to January 2021 [5]. Products without online nutrition information were excluded from analysis (*n* = 5 in 2017; *n* = 4 in 2020). Data for each product included serving size (g) and energy (kcal), saturated fat (g), total sugar (g), added sugar (g) (available in 2020 only), and sodium (mg) per serving. Using the serving size information, we calculated energy and nutrient content per 100 g. We also identified beverages that contained added sugar and/or NNS (i.e., sweetened beverages).

Data from the previous study included a measure of overall nutrition quality for food products, the Nutrition Profile Index (NPI) score, based on a nutrient profile model used to identify nutritionally poor foods and beverages that cannot be advertised to children under age 16 years under U.K. regulations [12]. This model had been used in previous studies to identify unhealthy food and beverage advertising in the United States [6, 13, 14], as well as a case study in New Zealand [13]. It assesses total energy, nutrients to limit (sodium, sugar, and saturated fat), and nutrients and food groups to encourage (fiber, protein and fruit, vegetable and nut content) per 100 g. NPI scores range from 0 (worst) to 100 (best). Foods that cannot be advertised to children in the U.K. have an NPI score of 62 or lower. To assess the healthfulness of drinks, we present energy and nutrients to limit (saturated fat, sodium and added sugar) as well as use of NNS (which are not recommended for children [15]).

We obtained 2021 advertising spending and gross rating points (GRP) data from Nielsen for all food and beverage products that were advertised on children’s TV channels (according to Nielsen’s classifications) in 2021. We then assigned each brand in the Nielsen advertising data (e.g., Pepperidge Farm Goldfish Crackers) to a product category using the CFBAI category definitions (e.g., savory snacks). We added one category (candy), which was not specified in the 2013 or 2020 CFBAI nutrition criteria. We also identified CFBAI-listed brands, defined as brands with any products on the CFBAI Product List as of August 2020 [14]. We analyzed brand-level advertising data as Nielsen data do not always indicate the individual product that was advertised (e.g., Goldfish Crackers – Cheddar or Pretzel). This analysis conservatively assumes that CFBAI companies only advertised CFBAI-listed products on children’s TV channels.

### Data analysis

All analyses were conducted using STATA 16.0 software [15]. Due to small numbers of CFBAI-listed products in some categories, we combined "Mixed dishes”, “Small meals” and “Main dishes and entrees” into one category (“Meals and entrees”) and “Cheese products”, “Nut butters” and “Waffles and pancakes” into one “Other” food category. CFBAI products identified as “Exempt beverages,” which the CFBAI defines as low-calorie, low-sodium beverages with ≤ 5 g added sugar per serving [11], were further classified into unsweetened waters and sweetened beverages, according to whether they contained added sugar or NNS. Approximately one-half of CFBAI product categories had no listed products, including milk and unsweetened water in 2017 and fruits and vegetables in 2020. In total, we examined six food (yogurts, sweet snacks, cereals, savory snacks, meals and entrees, and other) and four beverage categories (unsweetened waters, sweetened beverages, juices and milk) that had CFBAI-listed products in 2017 and/or 2020.

For each category, we calculated medians and interquartile ranges (IQR) for energy and nutrients to limit (saturated fats, sodium, and total sugars) due to non-normal data distribution. We examined total sugar because added sugar was not available in 2017 [16]. We then assessed differences in energy and nutrient content for each individual category and for all CFBAI-listed products combined between 2017 and 2020 using Wilcoxon rank-sum tests. For these tests, we defined statistical significance at *p* < 0.002 after applying a Bonferroni correction [17] for multiple comparisons (α = 0.05/30 tests).

Nielsen GRP data provide a per capita measure of advertisements viewed by all individuals in a demographic group over a period of time (by age group for 2021 in our data). We divided GRPs by 100 to obtain the average number of ads viewed on children’s TV channels in 2021 by all individuals in each age group [18]. We then summed ad spending and average number of ads viewed by children (ages 6–11 years) for each category, stratified CFBAI participating company (including CFBAI-listed and other products) versus other companies, and calculated proportions of total food and beverage advertising by category. In this analysis, we focused on children ages 6 to 11 years because CFBAI pledges at the time of data collection only covered advertising in media primarily directed to children under age 12, and most CFBAI companies pledged to not advertise any products in media primarily directed to children under 6 years [4].

Using the Nielsen data to identify brands with any advertising on children’s TV channels in 2021, we also calculated medians and IQRs for energy, saturated fats, sodium and added sugar, by category and in total for CFBAI-listed products advertised in 2021. To determine NPI scores by category, we obtained the median NPI score of all products analyzed in the category from the nutrition dataset [5]. We then compared differences for CFBAI-listed products that were advertised versus those that were not, via Wilcoxon rank-sum tests.

Finally, we compared the proportion of all CFBAI-listed products by category to the proportion of CFBAI-listed product ads viewed by children by category. To calculate median NPI score for all CFBAI-listed food products in ads viewed by children, we used the number of ads viewed to calculate the NPI score for products in 50% of ads viewed by children.

## Results

In both years examined, the categories with the most CFBAI-listed food products were yogurts (45% in 2017, 53% in 2020), sweet snacks (13% and 15%) and cereals (12% and 13%) (Table 2). A smaller proportion of meals and entrees were listed in 2020 (5%) versus 2017 (14%). The majority of CFBAI-listed beverage products in 2017 (70%) were sweetened beverages (fruit drinks or flavored waters), whereas most beverages listed in 2020 (60%) were unsweetened waters.

**Table 2:** Nutrient composition [median (IQR)] before and after Revised Nutrition Criteria

	**# of products**													
**Category**	**2017**	**2020**	**Energy (kcal)**		***p*-value**	**Saturated fat (g)**		***p*-value**	**Sodium (mg)**		***p*-value**	**Total sugars (g)**		***p*-value**
	**n(%)**	**n(%)**	**2017**	**2020**		**2017**	**2020**		**2017**	**2020**		**2017**	**2020**	
**All Foods**	258 (100)	202 (100)												
Yogurts	115 (45)	107 (53)	73 (53–80)	62 (53–75)	** < 0.001**	0.0 (0.0–0.0)	0.0 (0.0–0.9)	0.08	49 (43–53)	49 (37–53)	0.50	9.3 (4.7–11.5)	6.0 (4.7–9.7)	** < 0.001**
Sweet snacks	34 (13)	30 (15)	360 (348–381)	84 (78–110)	** < 0.001**	2.0 (0.0–3.6)	0.0 (0.0–0.0)	** < 0.001**	209 (109–357)	0 (0–16)	** < 0.001**	40.5 (37.0–50.0)	17.1 (14.2–21.7)	** < 0.001**
Cereals	31 (12)	27 (13)	393 (375–400)	389 (385–405)	0.57	0.0 (0.0–1.6)	0.0 (0.0–0.0)	0.17	517 (455–571)	528 (450–561)	0.87	31.3 (20.4–34.5)	30.8 (27.8–33.3)	0.95
Savory snacks	14 (5)	15 (7)	528 (490–581)	467 (467–467)	** < 0.001**	3.8 (3.5–4.5)	3.3 (3.3–3.3)	** < 0.001**	1018 (859–1126)	833 (800–867)	**0.001**	3.6 (0–4.5)	0.0 (0.0–0.0)	** < 0.001**
Meals and entrees^a^	36 (14)	10 (5)	108 (84–137)	117 (106–132)	0.78	0.9 (0.5–1.3)	1.1 (0.9–1.3)	0.43	220 (196–246)	214 (186–333)	0.63	3.4 (3.0–4.2)	3.1 (3.0–3.3)	0.58
Other categories^b^	28 (11)	13 (6)	286 (244–590)	286 (250–286)	0.48	9.4 (7.9–11.9)	9.4 (8.3–11.9)	0.74	667 (417–1098)	750 (619–1158)	0.43	9.4 (4.7–10.0)	5.3 (0.0–9.4)	0.17
**All Beverages**	50 (100)	43 (100)												
Unsweetened waters^c^	0 (0)	26 (60)		0 (0–0)	NA		0.0 (0.0–0.0)	NA		0 (0–0)	NA		0.0 (0.0–0.0)	NA
Sweetened beverages^c^	39 (78)	7 (16)	17 (2–17)	6 (0–6)	0.01	0.0 (0.0–0.0)	0.0 (0.0–0.0)	1.0	8 (0–8)	14 (0–14)	0.14	4.5 (0.0–4.5)	0.4 (0.0–0.4)	0.01
Juices	11 (22)	7 (16)	40 (40–45)	45 (28–51)	0.42	0.0 (0.0–0.0)	0.0 (0.0–0.0)	1.0	14 (11–17)	14 (11–14)	0.61	9.6 (4.0–13.9)	11.3 (5.6–11.3)	0.74
Milk	0 (0)	3 (7)		64 (59–64)	NA		0.6 (0.6–0.6)	NA		76 (55–76)	NA		9.3 (8.9–9.3)	NA

*IQR* Inter Quartile Range

^a^Includes CFBAI categories “Mixed dishes”, “Small meals” and “Main dishes and entrees”

^b^Includes CFBAI categories “Cheese products”, “Nut butters” and “Waffles and pancakes”

^c^These categories meet FDA regulations for “low calorie” (≤ 40 kcal) and “very low sodium” (≤ 35 mg), and contain ≤ 5 g added sugar per LSS (does not include diet sodas) and are exempt from CFBAI revised nutrition criteria

^d^*P*-value obtained from Wilcoxon rank-sum test, comparing energy and nutrients 2017 vs. 2020

### Nutrient content of CFBAI-listed products: 2017 versus 2020

There were some improvements in energy and individual nutrients in CFBAI-listed yogurts, sweet snacks and salty snacks from 2017 to 2020. Energy content per 100 g declined significantly (*p* < 0.001) for yogurts, sweet snacks and savory snacks. Saturated fat declined for sweet snacks and savory snacks, and total sugar declined for yogurts, sweet snacks and savory snacks. Median energy and total sugar content for sweetened beverages were also lower in 2020 compared to 2017 (*p* = 0.01). However, energy and individual nutrient content of CFBAI-listed cereals, meals and entrees, other food categories and juice did not change from 2017 to 2020.

### Advertising on children’s TV channels for CFBAI vs. non-CFBAI companies

In total, food and beverage companies spent $38.3 million to advertise on children’s TV channels in 2021, and on average children (6–11 y) viewed 256.2 of these ads (Table 3). Preschoolers (2-5y) viewed slightly fewer ads (253.8 ads), whereas adults (18–49 y) viewed one-third as many (79.0 ads), confirming that these ads were primarily targeted to children. The majority of food and beverage ads viewed by children (79%) were from CFBAI participating companies, of which nearly all (99.8%) were for brands with CFBAI-listed products.

**Table 3:** Food and beverage advertising on children's TV channels in 2021

Category	Ad spending^a^				Average # of ads viewed			
	Total ($000)	% of total	CFBAI companies ($000)	Non-CFBAI companies ($000)	6–11 y	% of total	CFBAI companies^b^	Non-CFBAI companies
Foods								
Cereals	$14,380.7	38%	$14,380.7	$0.0	110.3	43%	110.3	0.0
Meals and entrees	$6,068.6	16%	$6,064.9	$3.7	34.8	14%	34.8	< 0.1
Savory snacks	$4,423.1	12%	$4,423.1	$0.0	16.2	6%	16.2	0.0
Sweet snacks	$1,536.2	4%	$1,501.4	$34.8	11.8	5%	11.6	0.2
Yogurt	$675.7	2%	$675.7	$0.0	4.2	2%	4.2	0.0
Beverages								
Sweetened beverages	$2,652.4	7%	$2,651.3	$0.0	19.8	8%	19.8	0.0
Milk	$1,711.0	4%		$1,711.0	11.3	4%		11.3
Juices	$829.6	2%	$829.6	$0.0	5.9	2%	5.9	0.0
Other categories								
Candy	$4,113.9	11%	$0.2	$4,113.7	30.3	12%	< 0.1	30.3
All other^c^	$1,905.1	5%	$88.0	$1,295.3	11.6	5%	0.1	11.4
	$38,296.4		$30,615.0	$7,158.5	256.2		203.1	53.2

^a^Does not include advertising for restaurants. The following CFBAI food and beverage companies advertised on children’s TV channels in 2021: Kellogg, Kraft Heinz, Campbell, General Mills, Post Consumer Brands, Danone, Unilever, Keurig Dr Pepper, PepsiCo

^b^The 203.1 total ads by CFBAI companies includes 202.7 ads for listed brands and 0.4 ads for non-listed brands

^c^Includes gum/mints, baby food, energy drinks, regular soda, water, eggs, fruits and vegetables, condiments, cheese, meat and company-level ads

CFBAI companies had the highest amounts of advertising for cereal (ready-to-eat varieties) (43% of ads viewed), meals and entrees (14%), and fruit-flavored drinks (8%). They did not advertise any products in the milk and nut butter categories, despite listing products in these categories in 2020. Children also viewed food and beverage ads from non-participating companies, including for candy (12% of all ads viewed) and for healthier categories (e.g., milk, fruits and vegetables).

### Nutrition quality of CFBAI-listed products by children’s TV advertising status

Of the 245 products across 39 brands that CFBAI participating companies included on their lists of food and beverage products that may be advertised to children in 2020 (i.e., CFBAI-listed products), only 35% (*n* = 159) advertised on children’s TV channels in 2021**.** Moreover, median NPI scores were significantly lower (NPI = 47 vs. 72), and median energy, saturated fat and sodium per 100 g were significantly higher for CFBAI-listed food products that were advertised on children’s TV compared to products not advertised (*p* < 0.001) (Table 4). In addition, median energy and sodium content were higher for beverage products advertised on children’s TV channels than non-advertised beverages (*p* < 0.001) (Table 5).

**Table 4:** Nutrient composition^a^ of CFBAI-listed foods, by advertising status in 2021

Category	# brands (products)		NPI score		Energy (Kcal)		Sat. fat (g)		Sodium (mg)		Added sugar (g)	
	CTV^b^ ads	No CTV ads	CTV ads^c^	No CTV ads	CTV ads	No CTV ads	CTV ads	No CTV ads	CTV ads	No CTV ads	CTV ads	No CTV ads
Yogurts	1 (20)	5 (87)	71 (68–72)***	74 (72–76)	65 (55–71)	60 (53–75)	0.0 (0.0–1.0)*	0.0 (0.0–0.5)	49 (42–56)	48 (35–53)	5.3 (4.2–6.5)***	1.3 (0.0–6.5)
Cereals	8 (20)	5 (7)	46 (41–48)*	50 (44–54)	389 (381–406)	390 (389–405)	0.0 (0.0–0.6)	0.0 (0.0–0.0)	538 (494–564)***	444 (417–462)	30.8 (29.3–32.9)	30.8 (19.5–33.3)
Savory snacks	1 (15)	0	38 (36–44)		467 (467–467)		3.3 (3.3–3.3)		833 (800–867)		0.0 (0.0–0.0)	
Cheese	1 (10)	0	28 (22–32)		257 (237–286)		9.4 (7.9–11.9)		1143 (750–1158)		0.0 (0.0–0.0)	
Meals and entrees^d^	2 (8)	1 (2)	68 (64–69)*	72 (72–72)	130 (106–132)*	72 (69–75)	1.3 (0.9–1.4)*	0.4 (0.0–0.7)	199 (186–333)	242 (224–259)	0.0 (0.0–0.7)	1.7 (1.5–2.0)
Sweet snacks	1 (3)	2 (27)	32 (30–38)**	62 (60–64)	429 (393–429)**	82 (76–92)	5.4 (5.4–5.4)**	0.0 (0.0–0.0)	286 (286–286)**	0 (0–3)	28.6 (25.0–32.1)*	14.2 (13.3–17.2)
Nut butters	0	1 (3)		34 (34–38)		625 (609–656)		9.4 (9.4–10.9)		391 (313–406)		6.3 (4.3–6.3)
Total	14 (76)	14 (126)	47 (36–68)***	72 (64–76)	322 (81–429)***	68 (53–80)	1.1 (0.0–3.3)***	0.0 (0.0–0.0)	532 (56–785)***	43 (30–53)	4.3 (0.0–27.3)	2.7 (0.7–11.5)

^a^Values reported for energy and nutrients are medians and interquartile ranges per 100 g of product

^b^CTV: Children’s TV ads (advertised on children's TV channels)

^c^Asterisk denotes *p*-value obtained from Wilcoxon rank-sum test, comparing medians of products with child-directed ads versus no child-directed ads: * *P* < 0.05; **P < 0.01; ***P < 0.001

^d^Includes CFBAI categories “Mixed dishes”, “Small meals” and “Main dishes and entrees”

**Table 5:** Nutrient composition^a^ of CFBAI-listed beverages, by advertising status in 2021

Category	# of brands (products)		Energy (kcal)		Sat. fat (g)		Sodium (mg)		Added sugar (g)		Non-nutritive sweeteners	
	**CTV**^**b**^** ads**	**No CTV ads**	**CTV ads**	**No CTV ads**	**CTV ads**	**No CTV ads**	**CTV ads**	**No CTV ads**	**CTV ads**	**No CTV ads**	**CTV ads**	**No CTV ads**
Juice	1 (5)	1 (2)	51 (45–51)	27 (25–28)	0.0 (0.0–0.0)	0.0 (0.0–0.0)	14 (14–14)	11 (11–11)	0.0	0.0 (0.0–0.0)	No	No
Sweetened beverages^c^	2 (4)	2 (3)	6 (6–6)	0 (0–0)	0.0 (0.0–0.0)	0.0 (0.0–0.0)	14 (14–14)	0 (0–0)	0.0	0.0 (0.0–0.0)	Yes	Yes
Unsweetened water	1 (1)	3 (25)	0	0 (0–0)	0	0.0 (0.0–0.0)	0	0 (0–0)	0.0	0.0 (0.0–0.0)	No	No
Milk	0	1 (3)		64 (59–64)		0.6 (0.6–0.6)		76 (55–76)		4.2 (3.8–4.2)		No
Total^d^	4 (10)	7 (33)	26 (6–51)***	0.0 (0.0–0.0)	0.0 (0.0–0.0)	0.0 (0.0–0.0)	14 (14–14)***	0.0 (0.0–0.0)	0.0 (0.0–0.0)	0.0 (0.0–0.0)		

^a^Values reported for energy and nutrients are medians and interquartile ranges per 100 mL of product

^b^CTV: Children’s TV ads (advertised on children's TV channels)

^c^Nutrition information was not available online for Kool-Aid Jammers Zero Sugar and Capri Sun Reduced Sugar brands

^d^Asterisk denotes *p*-value obtained from Wilcoxon rank-sum test, comparing medians of products with child-directed ads versus no child-directed ads: * *P* < 0.05; **P < 0.01; ***P < 0.001

Individual food categories that had the lowest proportion of advertised CFBAI-listed products included sweet snacks (10%) and yogurt (19%), while categories with the highest proportion of advertised products included meals and entrees (80%), cereal (74%) and savory snacks and cheese (100%). For all individual food categories with some non-advertised brands, advertised products had significantly worse NPI scores than non-advertised products. Notably, advertised sweet snacks had a very low NPI score of 32, compared to 62 for non-advertised sweet snacks. For many categories, energy, saturated fat, sodium, and/or added sugar were also significantly higher for advertised compared to non-advertised products. Although none of the advertised beverages contained added sugar, four advertised sweetened beverage products contained NNS.

Table 6 compares the proportion of all CFBAI-listed products by category with the proportion of ads viewed by children (ages 6–11 y) on children’s TV channels in 2021 and the median NPI scores for all CFBAI-listed products versus advertised products. Although CFBAI-listed products in the yogurt category had the highest median NPI score (NPI = 74) and represented 53% of all CFBAI-listed food products, yogurts comprised just 2% of CFBAI-company ads that children viewed on children’s TV channels. In contrast, cereals represented 13% of all CFBAI-listed food products, but 62% of food ads from CFBAI companies viewed on children’s TV channels. Similarly, meals and entrees represented 5% of CFBAI-listed products but 20% of food ads viewed. Overall, the median NPI score of CFBAI-listed products in ads viewed by children on children’s TV was significantly lower than the median NPI score for all CFBAI-listed food products (NPI = 46 vs. 68).

**Table 6:** CFBAI listed products vs. products advertised on children’s TV channels in 2021 by category

	**Listed products**			**Advertised products**				
	# of brands (products)	%	Median NPI	# of brands (products)	%	# ads viewed by 6–11 y	%	Median NPI
Cereals	13 (27)	13%	46	8 (20)	26%	110.2	62%	46
Meals and entrees^a^	3 (10)	5%	68	2 (8)	11%	34.8	20%	68
Savory snacks	1 (15)	7%	38	1 (15)	20%	16.1	9%	38
Sweet snacks	3 (30)	15%	62	1 (3)	4%	11.4	6%	32
Yogurts	6 (107)	53%	74	1 (20)	26%	4.2	2%	71
Cheese	1 (10)	5%	28	1 (10)	13%	0.1	0%	28
Nut butters	1 (3)	1%	34					
**All foods**	**28 (202)**	**100%**	**68**	**14 (76)**	**100%**	**176.9**	**100%**	**46**
Sweetened beverages^b^	4 (7)	16%		2 (4)	40%	19.8	77%	
Juices	2 (7)	16%		1 (5)	50%	5.9	23%	
Unsweetened waters^b^	4 (26)	60%		1 (1)	10%	0.02	< 1%	
Milk	1 (3)	7%						
**All beverages**	**11 (43)**	**100%**		**4 (10)**	**100%**	**25.8**	**100%**	
**Total**	**39 (245)**			**18 (86)**		**202.7**^**c**^		

^a^Includes CFBAI categories “Mixed dishes”, “Small meals” and “Main dishes and entrees”

^b^These categories meet FDA regulations for “low calorie” (≤ 40 kcal) and “very low sodium” (≤ 35 mg), and contain ≤ 5 g added sugar per LSS (does not include diet sodas) and are exempt from CFBAI revised nutrition criteria

^c^This total does not include 0.4 ads for CFBAI company non-listed brands

Among beverage categories, unsweetened waters represented 60% of CFBAI-listed products but less than 0.1% of advertisements viewed on children’s TV channels, whereas sweetened beverages represented 16% of CFBAI-listed beverage products but 77% of beverage ads viewed. As noted earlier, no CFBAI-listed milk products were advertised on children’s TV in 2021.

## Discussion

The findings from this study expand upon previous research [5, 7, 19] showing that the CFBAI self-regulatory program continues to allow companies to advertise food and beverages in child-directed media that contradict nutrition education efforts. Our analysis found improvements in energy and/or nutrients of concern (saturated fat, sodium and/or added sugar) for products that “may” be advertised to children (i.e., CFBAI-listed products) in three food categories (yogurts, sweet snacks, and savory snacks) following implementation of CFBAI revised nutrition criteria in 2020. We also found that the overall nutrition quality of all CFBAI-listed beverages improved, including reductions in the proportion of beverages that contained added sweeteners (added sugars and/or NNS) and the addition of milk products, which were the only beverage products with added sugar in 2020. However, we also found limitations in the revised CFBAI nutrition criteria and that continued to allow nutritionally poor products to be advertised on children’s TV channels.

### Continued limitations in CFBAI revised nutrition criteria

Despite CFBAI statements that its nutrition criteria were revised to align with the 2015–2020 DGAs, we identified a number of criteria that did not align. For example, the 2015–2020 DGAs recommended limiting sodium intake to levels established by the Institute of Medicine (1,900 mg/day in 4–8 y; 2,200 mg/day in 9–13 y) [10]. Given the 1,500 kcal/day energy intake for an 8-year-old, the recommended maximum sodium/energy ratio would be 1.3 mg/kcal. Yet more than one-quarter (27%) of CFBAI listed food products exceed this ratio, including all listed cheese products (range 2.2 to 5.6 mg/kcal), meals and entrees (range 1.4–3.4 mg/kcal) and savory snacks (1.6–2.0 mg/kcal). The more recent DGAs (2020–2025) recommend even lower sodium intakes (1,500 mg/day in 4–8 y; 1,800 mg/day in 9–13 y).

Continued added sugar content in CFBAI-listed foods raises considerable concerns. Only 35% of U.S. children meet the recommended limit on added sugars (< 10% of daily calories [20]), and children consume on average 14% of total energy from added sugars [21]. However, CFBAI nutrition criteria allow cereal products to contain up to 12 g of added sugar per serving and up to 9 g for sweet snacks. In 2020, CFBAI-listed cereals contained a median of 31% added sugar by weight, with no improvement from 2017 to 2020. Total sugar in CFBAI-listed sweet snacks declined from 2017 to 2020, but median sugar content was 17% (primarily added sugar). Acceptable added sugar quantities set by CFBAI for these categories are also higher than limits set by non-industry entities for foods that should be advertised to children, including WHO Regional Office for Europe nutrient profile model (12.5 g/100 g for breakfast cereals and 0 g/100 g for sweet snacks) [22], the Chilean Food Labelling and Marketing Law (10 g/100 g) [23], and the Pan American Health Organization Nutrient Profile Model (≥ 10% of total energy from free sugars) [24].

Moreover, despite overall improvements in CFBAI-listed beverages, the nutrition standards still permit beverages containing NNS to be advertised in child-directed media (i.e., sweetened “exempt” beverages). This allowance contradicts recommendations by the American Academy of Pediatrics [25] and other nutrition and public health experts [26, 27]. Due to potential effects on sweet taste preferences, microbiome health, and other unknown longer-term health outcomes, NNS are not recommended for consumption by children [26, 27].

### CFBAI-listed products versus products advertised to children

Furthermore, CFBAI companies only advertised one-third of CFBAI-listed products on children’s TV channels in 2021, and the nutrition quality of advertised products was significantly worse than CFBAI-listed products that were not advertised on children’s TV. Some food categories were notable. Cereals represented just 13% of CFBAI-listed food products, but they contributed almost two-thirds of all CFBAI company food ads viewed by children on children’s TV channels in 2021, and these products had among the worst NPI scores of any category. In contrast, yogurt products had the highest NPI scores of any category in 2021, the nutrient content of CFBAI-listed yogurts improved from 2017 to 2020, and yogurts represented more than one-half of CFBAI-listed products in 2020. Yet ads for yogurt represented just 2% of CFBAI company food ads viewed by children on children’s TV. In the sweet snack category, only one of three brands on the CFBAI list was advertised on children’s TV in 2021, and these products had a very low NPI score of 32, compared to 62 for other (non-advertised) sweet snacks on the CFBAI list.

Beverage categories showed even greater discrepancies between the nutrition quality of all CFBAI-listed products and those that were advertised on children’s TV channels. In 2020, CFBAI companies included products that experts recommend for children (i.e., unsweetened water and milk) to the lists of beverages that may be advertised to children, and unsweetened waters represented 60% of CFBAI-listed beverage products. Nonetheless, unsweetened waters represented less than 0.1% of ads for CFBAI-listed beverage products viewed by children (6–11 years) on children’s TV, and not one CFBAI-listed milk product advertised on children’s TV. Moreover, beverages sweetened with NNS contributed more than three-quarters of CFBAI-company beverage ads viewed by children on children’s TV, despite representing just 16% of beverage products on CFBAI lists.

### Implications

Although CFBAI companies have added some healthier options to their list of products that “may” be advertised in child-directed media, they continue to permit child-directed advertising for food and beverages that are high in fat, sugar or sodium. At a minimum, the CFBAI nutrition criteria should align with nutrition standards established by the U.S. Department of Agriculture (USDA) for products that may be sold to children in elementary schools (i.e., Smart Snacks). These standards would require that foods advertised in child-directed media provide a meaningful contribution to a healthy diet (e.g., contain at least 50% by weight of a whole grain, fruit, vegetable) and would not allow any beverages that contain added sugar or NNS. Additionally, it would require products to have no more than 200 mg of sodium per serving sold, compared to the current CFBAI limit of 260 mg (savory snacks) and 260 mg (cereals). However, Smart Snacks standards continue to allow nutritionally questionable products such as whole grain Pop-Tarts or low-fat Cheetos [28]. Ideally, the CFBAI should follow the WHO recommendations and use a government-led nutrient profile model (such as the UK model used to calculate NPI score in our analysis) to classify foods that should not be marketed to children [3].

However, our findings suggest that CFBAI self-regulation is unlikely to lead to meaningful improvements in children’s diets and that allowing profit-driven companies to set their own nutrition standards is a key limitation of food industry self-regulation [29]. These findings also raise questions about companies’ true commitment to promoting healthier dietary choices for children, especially the finding that they continued to disproportionately advertise CFBAI-listed products with the worst nutrition profiles on children’s TV channels. The CFBAI’s focus on improvements in its nutrition criteria may provide companies with a public relations opportunity to claim they are addressing public health concerns about their marketing practices without requiring them to take meaningful action to improve the food environment for children. Similarly, global evaluations have shown that industry self-regulatory approaches are not effective at improving food marketing to children [3], but they do stave off effective mandatory regulations. Therefore, our findings also support WHO recommendations that governments in countries should implement and evaluate strong mandatory policies and legislation or laws that will protect children from powerful marketing of unhealthy food and beverages that threatens children’s health and wellbeing.

### Study limitations

This study is the first to examine changes in products advertised on children’s TV following the introduction of revised CFBAI nutrition criteria in 2020, but it has some limitations. First, we did not determine changes in nutrition components to encourage (e.g., vegetables, fruits, whole grains, under-consumed nutrients) as this information is not available on product packages. We did, however, utilize NPI score, which incorporated information about food groups to encourage in the model. In addition, we did not have information regarding the presence of NNS in foods (only beverages) in 2017, and therefore, could not compare these between both years. However, only three CFBAI-listed food brands contained NNS in 2020 (Danone Activia, Oikos and Light & Fit), and none of these brands advertised on children’s TV. Another limitation is that we present nutrition and advertising data for two points in time (2020 and 2021, respectively), and companies may have reformulated or introduced more nutritious products between both years. However our nutrition data was collected in November 2020 to January 2021, so we report the nutrition content of products at the beginning of the TV exposure data time period. In addition, 99.8% of CFBAI company brands that advertised in 2021 had CFBAI-listed products in 2020 and CFBAI nutrition criteria did not change during this time, so any improvements are likely to be minimal. Another limitation is that Nielsen data does not include children’s programming on other channels (for example, Saturday morning cartoons). However, in recent years broadcast channels in the US have moved away from children’s programming due to competition from children-focused satellite and cable channels, like Disney, Nickelodeon and Cartoon Network [30]. Finally, although we noted improvements in energy/nutrients of concern for three product categories, we cannot determine whether these changes were due to the new CFBAI nutrition criteria. Reductions in sugar could also have been due to the implementation of added sugar reporting on the Nutrition Facts label as required in January of 2020 [16].

## Conclusion

Although the nutrition criteria put forth by CFBAI to identify healthier products has improved slightly over the years, it has not resulted in substantial improvements in the nutrition quality of products advertised on children’s TV. Continued child-directed advertising of unhealthy food and beverages contradicts nutrition education efforts and public policies to promote healthy eating in children. If the CFBAI and participating companies wish to ensure that the products they advertise do not place children’s health at risk, additional improvements to CFBAI nutrition criteria and exclusion of unhealthy product categories are required. However, these findings add to the mounting evidence that food industry self-regulation is unlikely to lead to increased advertising of healthier dietary choices to children. As recommended by the WHO, governments in countries should implement and evaluate mandatory government legislation or laws for the regulation of unhealthy food marketing to protect children below the age of 18 years.

## Data Availability

The datasets generated and/or analyzed during the current study are not publicly available but will be made available upon request pending authorization from the senior author of the study.

## Abbreviations

CFBAI: Children’s Food and Beverage Advertising Initiative
DGAs: Dietary Guidelines for Americans
FDA: Food and Drug Administration
GRP: Gross rating points
NNS: Non-nutritive sweeteners
NPI: Nutrition Profile Index
TV: Television
U.K.: United Kingdom
U.S.: United States
